# “We were building the plane as we were flying it, and we somehow made it to the other end”: syringe service program staff experiences and well-being during the COVID-19 pandemic

**DOI:** 10.1186/s12954-022-00661-1

**Published:** 2022-07-15

**Authors:** Andrea Wang, Raagini Jawa, Sarah Mackin, Liz Whynott, Connor Buchholz, Ellen Childs, Angela R. Bazzi

**Affiliations:** 1 Boston University School of Medicine, Boston, MA USA; 2 Grayken Center for Addiction, Clinical Addiction Research and Education Unit, Section of General Internal Medicine, Boston Medical Center, Boston, MA USA; 3 Section of Infectious Disease, Boston Medical Center, Boston, MA USA; 4 Access, Harm Reduction, Overdose Prevention and Education (AHOPE), Boston, MA USA; 5 Tapestry, Springfield, MA USA; 6 Department of Community Health Sciences, Boston University School of Public Health, Boston, MA USA; 7 Abt Associates, Rockville, MD USA; 8 Herbert Wertheim School of Public Health, University of California, San Diego, 9500 Gilman Drive, MTF 265E (Mail Code 0725), La Jolla, CA 92161 USA

**Keywords:** COVID-19, SARS-CoV-2, Syringe service programs, Harm reduction work, Occupational health, Well-being

## Abstract

**Background:**

Syringe service programs (SSPs) provide essential harm reduction and prevention services for people who inject drugs in the USA, where SSP coverage is expanding. During the COVID-19 pandemic, US SSPs underwent unprecedented shifts in operational procedures (e.g., closures of physical sites, staff redeployment into pandemic response efforts). Given the critical role of US SSP workers in the pandemic, we sought to explore the occupational experiences and well-being of SSP staff to inform future emergency response efforts.

**Methods:**

From July–October 2020, we conducted semi-structured interviews with staff members of four SSPs in diverse regions of Massachusetts. Trained interviewers administered qualitative interviews virtually. Interviews were coded in NVivo v12 and thematic analysis identified common occupational experiences and related impacts on staff well-being in the context of the COVID-19 pandemic.

**Results:**

Among 18 participants, 12 (67%) had client-facing roles such as harm reduction specialists and six (33%) worked in program management or leadership. We found that staff were frequently anxious about SARS-CoV-2 transmission, which contributed to staff turnover. SSPs rapidly adapted and expanded their services to meet increasing client needs during the pandemic (e.g., food distribution, COVID-19 testing), leading to staff overexertion. Simultaneously, public health measures such as physical distancing led to staff concerns about reduced social connections with clients and coworkers. Through these challenges, SSPs worked to protect staff well-being by implementing flexible and tangible COVID-19-related policies (e.g., paid sick leave), mental health resources, and frequent communication regarding pandemic-related operational changes.

**Conclusion:**

SSPs in the USA adapted to the COVID-19 pandemic out of necessity, resulting in operational changes that threatened staff well-being. Despite the protective factors revealed in some narratives, our findings suggest that during prolonged, complex public health emergencies, SSPs may benefit from enhanced occupational supports to prevent burnout and promote wellness for this essential public health workforce.

## Introduction

Syringe service programs (SSPs) employ frontline, essential public health workers who provide critical harm reduction and prevention supplies and services to people who inject drugs. In the USA, where the coverage of SSPs is expanding nationally, supplies and services commonly offered include sterile syringes and injection equipment, overdose education and naloxone distribution, HIV and hepatitis C virus (HCV) testing, and referrals to on-site or external medical and substance use disorder treatment services [1]. In the USA and other countries in the past, large-scale public health emergencies have significantly disrupted SSP operations [2–4], likely contributing to increases in adverse health outcomes in surrounding communities [5, 6].

Most US SSPs remained open during the COVID-19 pandemic but reduced their hours of operation [7, 8] and experienced challenges related to staffing and supply chain shortages, loss of funding, and rapidly changing guidance from local governments and funders [8–10]. In response to COVID-19-related prevention recommendations and directives from federal and state agencies including the US Centers for Disease Control and Prevention (CDC) [11], most SSPs rapidly modified operations [9] to accommodate physical distancing [10, 12], expand existing or develop new outdoor and mobile delivery strategies, implement telemedicine [10, 13], pre-package harm reduction supplies for mailing to clients [7, 8], and increase and adapt community outreach strategies [12]. In line with these changes, many organizations prioritized the distribution of harm reduction supplies (e.g., syringes and sterile injection equipment) [9] over the provision of on-site prevention and clinical services including infectious disease testing and medications for opioid use disorder [7–10]. SSP staff experienced “task shifting” through which duties of healthcare workers were redistributed to SSP staff in order to maximize human resources for pandemic response efforts [14]. Specifically, new pandemic response roles for staff of many US SSPs included sourcing COVID-19-related information for clients [15] and providing SARS-CoV-2 testing to clients as well as surrounding communities [8].

Through the provision of essential public health services during the COVID-19 pandemic, SSP staff may have been susceptible to occupational burnout and stressors similar to those experienced among frontline healthcare workers, particularly during a time when public health and infection control recommendations were evolving [8, 9]. Even prior to the emergence of SARS-CoV-2, SSP staff were constantly exposed to poverty, drug use, and a high prevalence of drug-related overdose in the communities they served, leading to high levels of secondary traumatic stress, compassion fatigue, and burnout [16–20]. Emerging literature from the COVID-19 pandemic era has described changes in SSP operations and explored occupational burnout from the perspectives of non-SSP health workers; however, no studies to our knowledge have explored the impact of the pandemic on the well-being of the SSP workforce. Given the magnitude of the pandemic, the critical public health services delivered by SSPs in this context, and the increased risk of adverse mental health outcomes SSP staff uniquely face, we sought to understand how SSP staff perceive the pandemic to have impacted their occupational experiences and well-being. Understanding these experiences from the perspectives of a unique type of essential public health workers may help inform public health agencies’ future pandemic preparedness and emergency response.

## Methods

### Study design and sample

From July–October 2020, we conducted a qualitative study to explore the impacts of the COVID-19 pandemic on SSPs’ operational changes and staff experiences. We partnered with four geographically dispersed, community-based SSPs in Massachusetts that were sampled to represent different organization sizes, pre-pandemic modalities (e.g., fixed site, mobile delivery, or both), and geographic location (e.g., region, urbanicity). Four of six organizations we approached agreed to partner with us (1 declined participation and 1 did not respond, resulting in an organization-level response rate of 67%). SSP staff were eligible to participate if they were ≥ 18 years old and employed full time at an SSP. We first met with organization directors by videoconference to explain the study and gauge capacity and interest in participating and then invited three to five full-time staff members with diverse roles within their organizations (e.g., leadership, program management, direct service provision) to participate in one-time qualitative interviews. Eighteen of 21 individuals approached agreed to participate (3 declined or did not respond, resulting in an individual-level response rate of 87%). For interested individuals, trained interviewers then conducted eligibility screening, acquired informed consent, and conducted interviews via Zoom^©^ videoconferencing. All participants provided verbal informed consent.

### Data collection

Immediately prior to qualitative interviews, trained interviewers administered a brief verbal survey assessing participants’ professional roles, years at their current agencies, and total years of experience working with people who inject drugs and in HIV prevention or treatment. Interviewers then conducted in-depth qualitative interviews using a semi-structured interview guide with open-ended questions and detailed probes designed to elicit information about experiences working in harm reduction services before and during the COVID-19 pandemic. Examples of interview questions include: “Tell me about your professional experience working with people who inject drugs,” and “What concerns do you have for the safety or well-being of syringe exchange program staff?” Interviews lasted approximately 45–60 min, were audio-recorded using hand-held digital recorders, stored on a secure drive, and professionally transcribed following specific instructions regarding de-identification. Immediately following interviews, interviewers used a template with general prompts to help them write notes and reflections on key topics, new and emergent findings, and any other observations made during interviews. These notes helped informed discussion during weekly team meetings and the identification of emergent themes. Recruitment and interviewing continued until our study team determined through regular team meetings involving review and discussion of transcripts and interviewers’ notes that we had reached thematic saturation and that additional data collection would be unlikely to yield new or different insights on key topics of interest [21].

### Data analysis

We used a collaborative process to develop our codebook [22]. First, to generate preliminary deductive and inductive codes and related definitions, five study team members, including a lead investigator and four interviewers, independently reviewed the interview guide and several transcripts that were selected to represent different organizations and participant roles. We discussed and compiled these preliminary codes and definitions into a draft codebook that team members then independently tested by applying them to another set of transcripts (also selected to represent different organizations and participant roles). We then met to compare code application, discuss discrepancies and areas for revision, and modify the codebook for testing on another set of four transcripts. We continued refining codes and definitions through several additional rounds of this process until agreeing as a team on a finalized codebook. Two study analysts used NVivo (v12) to code the transcripts and met together weekly with the lead investigator to discuss coding progress and emergent findings. No further refinements to the codebook were made during this period.

For this analysis, we used a deductive and inductive approach to identify key themes after the preliminary coding described above was completed [23]. We first extracted data coded for “staff wellbeing” (which encompassed mental health, burnout, and staff turnover), “relationships at work,” “relationships outside of work,” “site characteristics” (which included site operations, internal structures, leadership, and policies), and COVID-19-related knowledge, testing, prevention, and infection. After an in-depth review of this data, the lead author developed and applied secondary codes relating to themes and subthemes relating to mental health (defined according to the World Health Organization’s definition of mental health as a “state of complete physical, mental, and social well-being and not merely the absence of disease or infirmity”; [24]), burnout (defined according to the WHO’s 11th Revision of International Classification of Diseases as a “syndrome resulting from chronic workplace stress that has not been successfully managed and is characterized by exhaustion, increased mental distance from one’s job, and negative feelings”; [25]), and specific occupational stressors that emerged in the data (e.g., concerns about occupational exposures to SARA-CoV-2). The lead author then conducted a closer reading of the data coded with these secondary codes to synthesize themes regarding occupational experiences and stressors during the COVID-19 pandemic. To illustrate key findings, we selected representative, anonymized quotes. As analyses identified similar experiences across client-facing and management/leadership roles, we did not stratify our presentation of findings but instead provided brief role descriptions along with quotes for additional context.

## Results

Among 18 participants representing four SSPs, 12 (67%) had client-facing roles as harm reduction specialists, counselors, and outreach workers, and 6 (33%) worked in program management and leadership (Table 1). Participants described numerous operational changes at their organizations as summarized in Table 2. From our thematic analysis, we identified six interrelated staff experiences during the COVID-19 pandemic, as detailed below.

**Table 1:** Characteristics of participating full-time staff of four syringe service programs in Massachusetts, July–October, 2020 (*n* = 18)

Participant characteristics	*n* (%) or median (IQR)
Job types/roles (not mutually exclusive)	
Director of organization or SSP	3 (17%)
Program coordinator or manager	6 (33%)
Harm reduction specialist, counselor, or outreach worker	12 (67%)
Other*	2 (11%)
Years working for current organization	1.8 (1.0–4.0)
Years working professionally with PWID	3.5 (1.0–8.5)
Years working in HIV prevention/treatment	3.0 (1.1–9.5)

*Recovery coach, alcohol/drug counselor, liaison to correctional facilities, administrative support

**Table 2:** Site characteristics and operational changes of four syringe service programs in Massachusetts, July–October, 2020

	Site A	Site B	Site C	Site D
City population	150,000–200,000	150,000–200,000	> 500,000	< 50,000
Time period of data collection	July 2020	July 2020	August–September 2020	October 2020
Full time operations	Yes	Yes	Yes	No (part time from March–April 2020)
Harm reduction services	Outdoor operations Online overdose prevention and remote naloxone kits assembly Increased community outreach and delivery of harm reduction equipment Temporary pause on post-overdose outreach until mid-July	Outdoor operations Increased community outreach and delivery of harm reduction equipment Use of mobile vehicle to facilitate community outreach Innovative client hotline to request for sterile supply delivery	Outdoor operations Increased community outreach and delivery of harm reduction equipment Innovative use of telemedicine for sterile supply and naloxone distribution	Outdoor operations until October Limited capacity available on-site Remote naloxone trainings continued for local organizations Innovative use of incentives to increase client uptake of naloxone and harm reduction trainings
Clinical services	Paused on-site testing of HIV and sexually transmitted infections Increased off-site testing of HIV and sexually transmitted infections Paused client education on safe injection practices Provided COVID-19 policy updates for detox facilities and social programs Continued provision of referrals and transportation to detox facilities (if available)	Paused testing of HIV and sexually transmitted infections Provided COVID-19 policy updates for detox facilities and social programs	Expanded community outreach to provide clinical consults, medications for HIV and opioid agonist therapy-used telemedicine innovations to continue clinical services remotely On-site clinic re-opened at limited capacity August 2020 Provided COVID-19 policy updates for detox facilities and social programs Continued provision of referrals and transportation to detox facilities (if available)	Paused on-site testing of HIV and sexually transmitted infections until October 2020
Miscellaneous programs	Expanded services to provide clients meals Snacks provided during outreach Food pantry operated outdoors	Expanded services to provide clients meals	Expanded services to provide clients meals	

### Concerns of occupational exposure to SARS-CoV-2

Participants consistently described concerns about occupational exposures to SARS-CoV-2, which were more pronounced earlier in the pandemic when much remained unknown regarding viral transmission and access to infection prevention supplies (including personal protective equipment [PPE] and testing) was inconsistent. Despite these concerns, participants continued to provide services to SSP clients including those involving close personal contact (e.g., drawing blood for infectious disease testing, responding to drug-related overdose). As one SSP program manager explained, “In the beginning, I think staff were certainly hesitant [about] knowingly putting yourself in danger. You were going to be around people for eight hours a shift…we weren’t even sure if we would have full PPE.” A harm reduction counselor similarly described uncertainty around workplace infection prevention measures, stating, “The [CDC’s] main concern was wearing masks and washing hands…The [clients] I work with haven’t showered in a week or two…how [are] we going to get prepared for that?” In addition to worrying about occupational exposures, participants were concerned about unknowingly exposing their family members with chronic health conditions to SARS-CoV-2, which one outreach worker described as “nothing to play with.”

Even following the implementation of refined, expanded infection prevention protocols, participants expressed concerns regarding SARS-CoV-2 outbreaks in local healthcare facilities or other organizations. As one program manager explained:

It’s stressful. [The local hospital] had an outbreak among employees and patients…That’s a huge organization with lots of policies and protocols in place. I mean, nobody’s walking out of this job, but it’s in our heads. Are we cleaning as much as we should? Are we doing enough? I think that we are. But it raises the question.

### Overextension from expanding services and roles

Interviewer notes documented significant staff turnover during our period of data collection, which may have been due to safety concerns as well as stress from increasing responsibilities and the early part of the “great resignation” of healthcare workers [26]. Among remaining staff members, this turnover led some participants to question how they would maintain basic operations. As one harm reduction specialist explained, “Our team has dwindled…people are quitting because of COVID. We’re typically a team of eleven and we’re functioning as team of five and a half right now. So, we’re definitely pushing it a little bit.”

As an essential public health service during the COVID-19 pandemic, SSPs were often asked to shift staff effort toward broad pandemic response initiatives and away from traditional SSP work. One outreach worker explained, “This [SSP program] is a two-to-three-person program that I do [myself]. It’s just me, basically. So we’re understaffed, underfunded, and undervalued.” Furthermore, in the context of the pandemic, many SSPs had reallocated some of their limited funding to obtain infection prevention supplies (e.g., PPE, hand sanitizer, cleaning supplies) and provide supplemental services (e.g., food distribution) to their clients. At the same time, given SSPs were essential public health providers serving specialized populations, some SSP staff experienced pandemic-related “task shifting” and were redeployed to SARS-CoV-2 testing efforts. A staff member explained that this redeployment of employees in state-funded organizations was part of an effort by the department of public health to quickly mobilize resources to increase low testing numbers in certain areas of the state. A program manager elaborated, being involved in new testing duties while wearing PPE was “very stressful for staff…[it was] hot and kind of scary. [Our director] is going to ask that we not test for a week just to give everybody a breather. We’re probably trying to accommodate too much, trying to be everything to everybody.” These experiences illustrate how some SSP workers may have felt over-extended and under-supported in maintaining core SSP operations while coping with redeployment into SARS-CoV-2-testing.

### Perceived reduction in ability to help clients

Related to the task shifting and new responsibilities described above, many participants described feeling less able to help the populations their organizations sought to serve. As testing efforts were originally focused on the general public, one SSP program manager described needing to caution their organization’s leadership about a potential gap in service provision, explaining that “we’re not [testing] the homeless or our SSP clients.” Other SSP staff also had to “advocate very sternly” to ensure equal access of new SARS-CoV-2 testing services.

In addition to concerns about new testing services not helping SSP clients sufficiently, participants also expressed concerns about how their elevated use of PPE and other COVID-19-related operational changes (e.g., delivering prevention supplies and services while maintaining physical distancing) interfered with their ability to reach and connect with some of the most disenfranchised SSP clients. One program coordinator described how transitioning to virtual prevention services and trainings may have excluded clients lacking consistent access to the requisite technologies: “If people can go to a Narcan training on Zoom, for the most part, they have some level of privilege.” Some participants also shared concerns that interfered with their ability to build rapport and trust with SSP clients. When wearing heavy PPE (e.g., face and eye protection, full body coverings, gloves) and interacting with clients, one outreach worker explained how wearing full PPE symbolized the very medical establishment that misunderstood and underserved their clients:

It was a lot harder to connect with my clients because I like to get on a personal level and just talk to them like they’re my friends. The PPE made it more intimidating for people. It was harder for them to see me as an outreach worker. Now I was like this medical person who probably doesn’t know anything about addiction.

Participants also shared their observations of how the pandemic significantly disrupted their clients’ access to substance use disorder treatment and social services. Furthermore, their clients were largely unable to maintain physical distancing and engage in other recommended prevention behaviors. Witnessing clients in this state of crisis without being able to directly change these social and structural factors led some participants to feel moral distress, guilt, and powerlessness, as explained by one program manager who was also involved in direct service provision:

There was only so much we could do to help…[We used to] be able to get a lot of people into detoxes [but during COVID-19] they weren’t taking significant numbers…Then there’s also the whole component of seeing your clients just degrade over the weeks because they don’t have any place to go. Or you’re hearing them cry because they don’t have enough money...Helping people before meant you could do so much, and then COVID happens and all you can do is give them a bottle of water and a granola bar. It’s very difficult to reconcile with yourself.

These narratives demonstrate how pandemic-related operational changes led to concerns about reduced ability to serve SSP clients, leading to frustration, reduced morale, and a sense of futility in participants’ work.

### Isolation in the workplace

Many participants described how emotional support for SSP workers prior to the pandemic involved team cohesiveness, peer support, and the general ability to share experiences and debrief with coworkers. However, some participants, including a harm reduction specialist, noted how operational changes made connecting with coworkers increasingly challenging:

[Our SSP] is a team. Before COVID, it was like family: laughing, talking, everybody knows everybody, and in fact, everybody knows everybody’s business. But when COVID hit, it was like, scattered…a different atmosphere…The closeness of our team is coming apart because we’re not all together; we don’t see each other all the time.

Participants also described reduced personal interactions with SSP clients, which had provided them with an important source of connection and job satisfaction before the pandemic, as another outreach worker explained: “People [would] come in, I’d talk to them, ask how their day is, and that’s when people let out everything…That’s one of the main things I miss, the intimate conversations…I used to learn a lot from [them].”

Contrary to above, a participant from one organization described how the pandemic led them to have a “tighter, more cohesive” team in which staff members “look[ed] out for one another.” A harm reduction specialist detailed how the shared experience of working during a public health crisis created opportunities for connectedness:

Our team has definitely been stretched in a lot of different ways. Sometimes there’s more tension than there usually is. Sometimes there’s more comradery than typical. Some of us have come to rely on each other even more than usual, just trying to support each other and [there’s] really a lot of collaborative teamwork.

### Added meaning to work

While most SSP staff felt frustration and less able to help their SSP clients, others found that being engaged in innovative and necessary harm reduction and public health work added meaning to their jobs. One harm reduction counselor described improving outreach to clients by distributing water to reduce the risk of dehydration, explaining that “[if] a client is going to shoot up and they’re dehydrated, they’re going to overdose…little things we’re doing like that really mean a lot.” Increased use of texting with clients was described as resourceful and motivating for participants and their coworkers as it created a “more casual relationship that feels more natural” with clients, helping to maintain staff connectedness to clients and increase accessibility to SSP services. A program director echoed “how darn creative we had to get, and how quickly we adjusted…We needed cloth masks to give to people, and people started sewing…It was great. We were making it up as we went. We were building the plane as we were flying it, and we somehow made it to the other end.”

Many participants described feeling a strong sense of responsibility to step into new roles (e.g., SARS-CoV-2 testing), knowing that they were the sole frontline providers for their clients; as one outreach worker asked, if they “backed out, who’s there to do it?” This participant then went on explain, “We were already in [it] with the opiates and the overdoses, and then we get hit with a pandemic. So, there was no way we were going to close our doors…That’s the bottom line.” Participants felt a sense of duty and commitment to sustain their harm reduction service delivery to clients even while feeling taxed and fulfilling new roles because they feared that their clients would be abandoned otherwise.

### Positive workplace adaptations

Despite the numerous challenges to participants’ well-being during the COVID-19 pandemic, many also discussed the positive impacts of innovative operational changes that increased their comfort, motivation, and satisfaction at work. First, participants cited their appreciation for transparent communication from organizational leadership regarding rapidly changing guidelines and resources of PPE, which helped them feel safer. A harm reduction specialist described how their organization “has really done the best they can in keeping things flowing efficiently….Our director has [been] as transparent as possible [in] sharing what she knows.” Similarly, a program manager from a different organization described strengths in their director’s approach: “As soon as he heard [from upper administration], he informed us as to what the changes were going to be, what we were going to do, and the projection of how long this would be going on.” As one program director noted, in the context of concerns about a mounting “second surge” in COVID-19 cases, “we have PPE to get through six months, which is awesome.”

Second, participants described the importance of innovative measures to support their physical and mental health. In terms of their physical health, participants at one site described how new job duties related to COVID-testing were compensated in the form of “hazard pay,” while “COVID-time” (i.e., paid sick leave) at another site helped staff feel supported and empowered to prioritize their physical health without feeling pressured “to come to work even though they’re sick.” In terms of staff mental health, participants appreciated an increased frequency of “wellness checks” between managers and staff, and the promotion of taking time off, as a manager explained: “Nobody’s been denied vacation time. Or somebody will say, ‘You know, I just need a mental health day off,’ and it’s just like, ‘Sure, absolutely understandable,’ because it’s tough.” Generally, organizations’ leadership acknowledged how job stress was exacerbated by the pandemic and promoted staff wellness checks and time off, leading participants to feel supported. Conversely, a program manager at one SSP did not observe these adaptations and worried about staff well-being within their organization:

The thing that has felt the least supportive is just the sheer amount of chaos and feeling like I have to figure certain stuff out for myself. There are definitely people at my work who are supportive who check in and make sure that we’re doing okay, but I think that sometimes, certain service programs or non-profits in general are not the most supportive places to work.

## Discussion

As providers of essential public health services, US SSPs underwent numerous operational changes during the COVID-19 pandemic to deliver critical harm reduction and COVID-19-related services to their clients. SSP staff, a unique segment of the US public health workforce, have been instrumental in carrying out pandemic response efforts but may experience elevated risk for occupational burnout. Prior to the pandemic, SSP staff had been repeatedly exposed to secondary traumatic stress, compassion fatigue, and burnout from working in the context of the opioid overdose epidemic [16–20]. Many SSP staff have a history of substance use or identify as being “in recovery” [1] and are intimately connected to the communities they serve [27–31]. As evident in our data, this connection contributed to a sense of dedication in serving clients during the pandemic but may have also amplified adverse impacts on their own well-being. These adverse mental health outcomes may threaten job satisfaction, morale, productivity, staff retention, and even the quality of care provided to clients [32–35]. Indeed, in our sample of SSP staff across Massachusetts, we found that COVID-19-related programmatic adaptations led to numerous emotional and occupational stressors for staff. To our knowledge, this is one of the first in-depth studies of how COVID-19 has impacted the SSP workforce, and our findings carry important implications for ongoing and future pandemic preparedness efforts within this sector of the public health workforce in the USA and potentially beyond.

Recent research by Wenger et al. highlighted the ingenuity of US SSPs in effectively adapting their programs to the demands of the COVID-19 pandemic while facing limited funding and staffing and supply chain challenges; however, the authors questioned the sustainability of relying on SSPs to innovate with limited resources [9]. Indeed, individuals employed in this sector demonstrated tremendous dedication to meeting clients’ growing needs but sometimes at the cost of their own well-being [36, 37]. Based on the Occupational Health and Safety Administration classifications, SSP workers would be considered at high to very high risk of SARS-CoV-2 exposure [38]. However, unlike other industries that possess robust occupational health infrastructures, most SSPs are community-based non-profit organizations. In this context and as has been in among other frontline healthcare workers, it is unsurprising that SSP workers in our study cited SARS-CoV-2 exposure risk as a significant source of occupational stress, particularly as their organizations scrambled to re-allocate funding to support infection prevention protocols amidst acute PPE shortages and funding constraints [39]. Furthermore, information regarding procedures to operate safely were difficult and sometimes impossible to find. Both Wenger et al. and Glick et al. highlighted the lack of guidance from parent organizations, leaving SSPs to improvise programmatic changes from general CDC guidelines which were themselves difficult to interpret [8, 9]. SSP workers who experienced task shifting or redeployment into COVID-19-related roles by the state department of public health, while critical to pandemic response efforts, may have also experienced frustration and moral distress, which has been linked to reduced job satisfaction and retention [40, 41]. Another job adaptation, wearing full PPE, also added to occupational distress as staff were concerned about their appearances becoming overly “medicalized.” This could symbolize medical institutions that have historically stigmatized people who inject drugs, creating disconnection between staff and clients. In light of the literature highlighting the social constructions of illness [42], staff may have felt as though they were placing a label of “illness” on their clients, which clients had not chosen or did not necessarily agree with. Finally, multiple studies of healthcare workers following public health emergencies have identified post-traumatic stress disorder, lasting burnout, and elevated staff turnover as consequences [43]. Unlike other health services occupations, SSP staff are often the only prevention service providers with whom SSP clients have regular contact [44], filling a critical service gap for the socially and structurally marginalized populations they serve.

In order to ensure that the SSP workforce is sustained during public health emergencies like COVID-19 pandemic, we have highlighted some organizational and policy-level supports that should be established to promote staff resilience and negate burnout (Table 3). Given that SSP staff have a wide variety of roles and training backgrounds, federal and state agencies should find ways to offer tailored technical assistance for implementation of infection control protocols to SSP staff so they can safely, effectively, and sustainably transition into new clinical roles and expand their existing roles. An example of key guidance includes technical recommendations regarding high-risk encounters such as naloxone administration and using bag valve masks. There should be increased transparency from policymakers about shifting SSP staff to other public health activities, and SSP staff at all levels should be engaged in the decision-making processes for task shifting to promote organizational and individual staff buy-in and self-efficacy. While providing harm reduction services, SSPs should ensure reliable streams of PPE and other hygiene equipment to reduce risk of occupational exposure to SARS-CoV-2. Lastly, supplementary funds should be provided to SSPs to support their increased infection control procedures, service expansion efforts, and increased use of technology in virtual care delivery. Particularly since there is a lack of overlapping services and resources available for people who inject drugs, any public health initiative that involves task shifting of SSP staff should ensure there is adequate staffing within SSPs so that core harm reduction services are not interrupted and there is no strain for service provision on existing staff. Since SARS-CoV-2 infection control protocols resulted in many SSPs reducing their clinical services, policymakers and SSPs should collaborate to develop strategies to reduce service gaps for SSP clients. Strategies may include creating new accessible low-barrier clinical sites, increasing mobile clinical service delivery, or providing SSPs resources to develop alternative program adaptations. Finally, if SSP staff roles are modified or expanded, it may be necessary to allocate time for hiring, training, and capacity-building into timelines for implementing new services, and to re-evaluate staff compensation to fairly reimburse increased workload and occupational risk.

**Table 3:** Challenges to SSP staff well-being during public health emergencies and corresponding strategies to promote well-being

Challenge	Strategies to promote SSP staff well-being
Concerns of occupational exposures due to the emergency	Policy recommendations:
	Provide SSP staff with tailored technical assistance and training to implement modified and new services (e.g., administering naloxone, new infection control measures and testing services)
	Guarantee SSPs a steady supply of safety equipment (e.g., PPE and personal hygiene supplies) for staff and clients
	Ensure sufficient funding sources to cover any changes in operations and other necessary emergency-related purchases
	Provide staff hazard pay for expansion of responsibilities and work during an emergency
	Consider need to supplement technology resources for clients and staff to support virtual care delivery
	Organizational-level recommendations:
	Leaders should be more visible and accessible to staff, and provide transparent, frequent communications regarding the emergency and related operational changes
Overextension from expanding services and roles	Policy recommendations:
	Increase transparency about shifting of SSP staff to other public health activities and involve all levels of SSP staff in organizational decision-making
	Initiatives involving task shifting of SSP staff should consider the adequacy of staffing within SSPs to maintain uninterrupted core harm reduction service delivery and low stress on remaining staff members
	Consider capacity-building (hiring, training, improvising program adaptations) in the timeline of implementing new service
	Ensure that funding to SSPs reflect any increase in the breadth of service provision and re-evaluate staff compensation to reflect increase in responsibilities
	Organizational-level recommendations:
	Create an environment conducive to talking about fears, burnout openly (establish a culture of taking breaks and sick leave, implement an “open door” policy)
	Provide regular psychological care and mental health monitoring through counseling and check-ins with management
	Consider using longer interventions and online platforms in operationalizing mental health support; screen mental health of staff to identify individuals with the greatest need for targeted support delivery
Perceived reduced ability to help clients	Policy recommendations:
	Collaborate with SSPs to reduce service gaps for SSP clients by implementing strategies such as creating new accessible low-barrier clinical sites, increasing use of oral point of care HIV and STI testing, increasing mobile clinical services, or providing SSPs resources to develop alternative program adaptations
	Organizational-level recommendations:
	Include SSP staff in decision-making, particularly staff involved in direct service provision
	Implement interventions targeting moral distress such as education, and staff reflection and discussions on moral distress
Workplace isolation	Organizational-level recommendations:
	Organize regular team-building exercises such as team huddles and virtual activities over videoconference
Adding meaning to work	Organizational-level recommendations:
	Recognize and appreciate staff work
	Minimize role ambiguity and role conflict

SSP workers on the frontlines of the two public health crises (COVID-19 and opioid overdose) should have robust access to mental health resources and interventions to address the inherent occupational stress and anxiety they face. Prior studies evaluating the mental health of physicians showed that interventions done at the organization-level were more effective in improving outcomes than those done at the individual-level [45]. Similarly, participants in our study described how organization-level policies implemented by their SSP managers and director benefited their mental health (e.g., normalization of taking breaks, paid sick leave, implementing “open door” policies to increase the accessibility of management and leadership, and providing regular psychological care). In addition, SSPs could aim to reduce staff moral distress with interventions such as normative staff education about moral distress and encouraging reflective debriefing about workplace experiences [46, 47]. Strategies have been developed to decrease symptoms of secondary trauma including education, self-efficacy interventions, maintaining self-care, developing anxiety-reduction skills and having manageable workloads [48–50]; however, evidence on the efficacy of these interventions is limited [51]. Lastly, to aid SSP staff in finding meaning in new roles, SSP leadership can recognize the importance of staff work and minimize role ambiguity and role conflict, strategies which have been shown to increase staff motivation and work satisfaction [52–54]. In considering the operationalization of these interventions, utilizing online platforms offers the benefit of increasing flexibility for staff to engage with support, while longer interventions and targeting support for frontline workers who are at greatest need through mental health screening may be effective strategies [55]. SSP leadership should increase their presence among their workforce and provide frequent and transparent channels of communications to ameliorate risk of anxiety around operational changes. Lastly, some participants described difficulty staying physically and emotionally connected with coworkers due to operational changes. SSP leadership should leverage the fact that social support is protective of staff well-being [56–62] possibly by organizing regular team-building exercises such as in-person “team huddles” and virtual activities leveraging videoconferencing technology. Furthermore, coordinated responses such as assembling supplies to address supply chain challenges could potentially build team solidarity and moral.

There are several limitations of our study. First, our findings are based on interviews with SSP staff that were done at a single point in time over a four-month period (July–October 2020) during the early months of the COVID-19 pandemic. Additional longitudinal research is needed to evaluate longer-term impacts of the pandemic on SSP staff and should include staff in a diversity of roles and organizations of varying sizes and types. Second, we did not formally assess specific mental health or occupational burnout outcomes, though our findings suggest a need for further investigation within this population and setting, including through larger quantitative studies using validated measures. Third, this research was based in Massachusetts, a state with robust public health infrastructure and a generally strong political and funding support for harm reduction programming. As all four organizations included in our study received State support for their programming (including new COVID-19-related services), our findings may not be transferable to SSPs operating more independently from state or local health departments; future studies should investigate staff experiences and outcomes in other US regions and countries globally where policies, funding, and political support for harm reduction may differ. Finally, as abundant evidence demonstrates that the pandemic disproportionately impacted Black and Latinx communities, research with larger sample sizes should explore differences in SSP staff experience by race and ethnicity in order to better inform programmatic and policy decisions. To maintain staff and client privacy, we chose to omit data on the racial, ethnic, and gender of participants given the small size of our sample and location within a small geographic area and close network of organizations; however, this results in an important limitation. Furthermore, we restricted our sample to full-time SSP staff; additional research is needed to understand the occupational stressors that may have been experienced by part-time and volunteer SSP workers. Despite these limitations, this study was the first, to our knowledge, to investigate SSP staff perspectives, personal experiences, and well-being in the context of the COVID-19 pandemic.

## Conclusions

In the face of challenging circumstances during the unprecedented COVID-19 pandemic, SSPs adapted quickly and effectively to maintain the provision of their staple harm reduction services while also contributing to pandemic response efforts. While SSP staff and leadership should be commended for the incredible efforts undertaken during this period, our study identified the presence of worrisome factors that could negatively impact SSP staff well-being, including risk of occupational exposure to SARS-CoV-2, overexertion driven by funding, staffing, and supply shortages, task shifting into new COVID-19-related responsibilities, moral distress, and workplace isolation, all of which could place staff at elevated risk of burnout. Based on these findings, we argue that adequate supports must be available to promote SSP worker well-being and retention and sustain positive work environments in the pandemic context and beyond. Additional research and programmatic efforts engaging this population of essential public health workers could help inform future pandemic preparedness efforts in the USA and possibly other contexts.

## Data Availability

The datasets used and/or analyzed during the current study are available from the corresponding author on reasonable request.

## Abbreviations

CDC: Centers for disease control and prevention
COVID-19: Coronavirus disease 2019
HCV: Hepatitis C virus
HIV: Human immunodeficiency virus
PPE: Personal protective equipment
PWID: People who inject drugs
SARS-CoV-2: Severe acute respiratory syndrome coronavirus 2
SSP: Syringe service program
US: United States
